# The ELK3-GATA3 axis orchestrates invasion and metastasis of breast cancer cells *in vitro* and *in vivo*

**DOI:** 10.18632/oncotarget.11427

**Published:** 2016-08-19

**Authors:** Sun-Young Kong, Kwang-Soo Kim, Jiewan Kim, Min Kyeong Kim, Ki Hong Lee, Je-Yong Lee, Nuri Oh, Ji-In Park, Ji-Hoon Park, Sun-Hee Heo, Sung Han Shim, Dong Ryul Lee, Keun Pil Kim, Kyung-Soon Park

**Affiliations:** ^1^ Department of Biomedical Science, College of Life Science, CHA University, Seoul, Korea; ^2^ Department of System Cancer Science, Graduate School of Cancer Science and Policy, National Cancer Center, Seoul, Korea; ^3^ Translational Epidemiology Branch, Research Institute, National Cancer Center, Seoul, Korea; ^4^ Department of Laboratory Medicine, Center for Diagnostic Oncology, Hospital, National Cancer Center, Seoul, Korea; ^5^ Biomedical Research Center, Asan Institute for Science and Department of Pathology, Asan Medical Center, University of Ulsan, College of Medicine, Ulsan, Korea; ^6^ Department of Life Science, Chung-Ang University, Seoul, Korea

**Keywords:** ELK3, GATA3, metastasis, migration, invasion

## Abstract

Triple-negative breast cancer is a highly aggressive tumor subtype that lacks effective therapeutic targets. Here, we show that ELK3 is overexpressed in a subset of breast cancers, in particular basal-like and normal-like/claudin-low cell lines. Suppression of ELK3 in MDA-MB-231 cells led to transdifferentiation from an invasive mesenchymal phenotype to a non-invasive epithelial phenotype both *in vitro* and *in vivo*. Suppression of ELK3 resulted in extensive changes in genome expression profiles. Among these, GATA3, a master suppressor of metastasis, was epigenetically activated. Also, suppression of GATA3 led to the restoration of migration and invasion. These results suggest that the ELK3-GATA3 axis is a major pathway that promotes metastasis of MDA-MB-231 cells.

## INTRODUCTION

Up to 90% of deaths in patients with solid tumors can be attributed to metastasis [1]. The metastatic cascade is a complex process that culminates in tumor cell acquisition of genetic and epigenetic changes that enable them to survive the journey from the primary tumor site to the metastatic site. Aggressive tumor cells are predicted to display a high degree of cellular plasticity, meaning that they display both epithelial and mesenchymal characteristics. Recent studies identified a set of pleiotropic transcription factors (Snail, Slug, and ZEB1/2), which together form an intricate transcriptional circuit to induce epithelial-mesenchymal transition (EMT) [2, 3]. Although several transcription factors and extracellular factors that control EMT and MET have been identified, the underlying molecular mechanisms remain unclear.

ELK3 is an ETS domain-containing protein capable of forming a ternary complex with DNA and Serum Response Factor. Although ELK3 functions as a transcriptional repressor, it can be transformed into a transcriptional activator via RAS/ERK signaling [4]. Another interesting feature of ELK3 is that it contains a leucine-rich nuclear export signal within the first helix of the ETS DNA-binding domain, which is involved in the export of ELK3 from the nucleus to the cytoplasm in response to stress activated kinases, in particular JNK [5].

It is unclear whether ELK3 functions as a tumor promoter or as a tumor suppressor. Tumors that develop in mice lacking the ELK3 protein are small due to a lack of vascularization/oxygenation [6, 7]. Malignant progression of squamous cell carcinoma (SCC) requires ELK3 expression, and knockdown (KD) of ELK3 severely impairs tumor growth and inhibits progression from benign papillomas to SCC [8]. Conversely, ELK3 also functions as a tumor suppressor. The oncogenic microRNA, has-miR-155-5p, targets ELK3 and is implicated in the dynamics of the hypoxic response, which is a critical cellular process in cancer [9]. ELK3 strongly represses transcription of the proto-oncogene, c-fos, and overexpression of ELK3 inhibits the proliferation of pancreatic cancer cells [10]. Furthermore, expression of caveolin-1, which accumulates in high amounts in metastatic lymph nodes during human lung tumorigenesis, is suppressed by ELK3 [11].

Breast cancer is the second leading cause of cancer-related death in women, and its incidence increases with age. Breast tumors can be classified as distinct subtypes based on gene expression profiling and hierarchical clustering analysis [12]. Basal triple-negative breast cancer (BTNBC), which lacks estrogen receptor (ER), progesterone receptor, and ErbB2 expression, has a poor outcome and high metastatic potential. The human breast cancer cell line, MDA-MB-231, recapitulates many of the biological and molecular features of BTNBC, and has been used extensively as a model cell line to study the molecular mechanisms underlying metastasis.

GATA3 is a zinc-finger transcription factor that is involved in development and differentiation. Recent studies show that GATA3 is exclusively expressed in ER-positive, well differentiated, early stage breast cancers [13]. In addition, expression of GATA3 inhibits breast cancer metastasis by driving invasive cancer cells to undergo MET [14]. GATA3 nucleates a transcription repression program to inhibit the invasive potential of breast cancer cells *in vitro* and to suppresses metastasis *in vivo*; it does this by targeting a cohort of genes, including ZEB2, which is critically involved in EMT [15]. However, the tumor suppressor activity of GATA3 has been challenged by recent reports showing that GATA3 is similarly expressed in both primary and metastatic breast carcinoma [16, 17].

Previously, we reported that ELK3 is highly expressed in MDA-MB-231 cells, and that it plays a positive role in metastasis [18]. The aim of the present study was to increase our understanding of the mechanism underlying ELK3 activity during tumorigenesis and metastasis of BTNBC. We provide evidence that ELK3 is an upstream inhibitor of GATA3. The *in vitro* and *in vivo* results suggest that suppression of ELK3 reprograms metastatic mesenchymal MDA-MB-231 cells into a non-metastatic epithelial cancer cell type. Epigenetic activation of GATA3 in ELK3 KD cells, and restoration of migration and invasion ability upon suppression of GATA3 in ELK3 KD cells, suggests that the ELK3-GATA3 axis is a major pathway that activates metastasis of MDA-MB-231.

## RESULTS

### Suppression of ELK3 *in vitro* reprograms MDA-MB-231 cells to a less invasive phenotype

**Figure 1:**
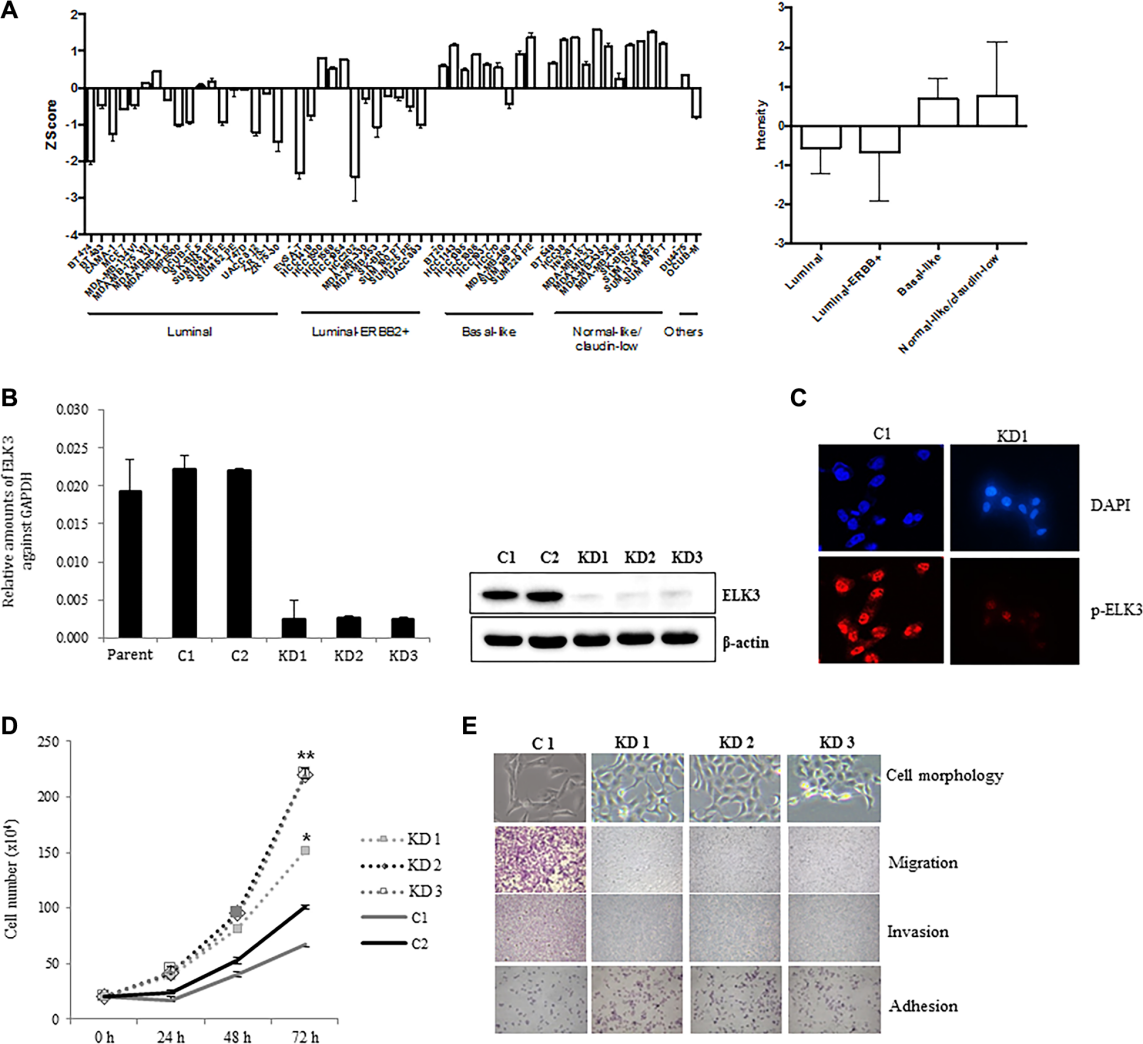
ELK3 regulates migration and invasion of MDA-MB-231 cells *in vitro* (**A**) ELK3 expression in 51 breast cancer cell lines (18 luminal, 12 luminal-ERBB2+, nine basal-like, 11 normal-like/claudin-low, and one other) was analyzed. Data were obtained from public Micro Array databases (GEO: GSE41313; [19]) (left panel), and the average for each group was calculated (right panel). (**B**) MDA-MB-231 cells were transduced with a retrovirus expressing shRNA specific for ELK3, and three stable cell lines (KD1, KD2, and KD3) were selected following analysis of ELK3 expression. Control cell lines (C1 and C2) were generated by transduction of a control retrovirus. (**C**) Phospho-ELK3 localized in the nucleus of C1 and KD1 cells, as detected by immunocytochemical staining. (**D**) Proliferation of C1, C2, KD1, KD2, and KD3 cells was analyzed by counting cells at the indicated times after seeding. (**E**) Cell morphology was observed under a light microscope. Migration, invasion, and adhesion were analyzed as described in Materials and methods. Error bars represent the standard error from three independent experiments, each performed using triplicate samples. *P < 0.05 and **P < 0.01 (Student's *t*-test).

We first examined ELK3 expression profiles in a cohort of 51 molecularly well-characterized human breast cancer cell lines [19]. The 51 cell lines were grouped into the following types: luminal, luminal-ERBB2+, basal-like, and normal-like (claudin-low). Of the genome expression profiles obtained by microarray analysis (all of which are deposited in the Gene Expression Omnibus data repository [GEO: GSE41313]), we compared that of ELK3 between the four breast cancer subgroups. Figure 1A shows that ELK3 expression in basal-like and normal-like/claudin-low cell lines was higher than that in luminal and luminal-ERBB+ cell lines. Notably, MDA-MB-231 was the top-ranked cell line in terms of high ELK3 expression. Stable shRNA-mediated KD of ELK3 was established in three MDA-MB-231-GFP-Luc cell lines (KD1, KD2, and KD3) (Figure 1B). Immunostaining with a phospho-ELK3 antibody revealed that phospho-ELK3 localized in the nucleus of control MDA-MB-231 (C1) cells but not in ELK3 KD cells (Figure 1C). The first feature we noticed in ELK3 KD cells was a marked increase in the proliferation rate (Figure 1D). Since this suggested that the tumorigenicity of MDA-MB-231 cells was increased by suppression of ELK3, we next examined other parameters linked to breast cancer progression. The critical characteristics of metastatic cancers are mesenchymal cell morphology, high migration and invasive capacity, and loss of adhesion to the underlying basement membrane, which allows invasion into surrounding tissues or the circulatory system. Contrary to our expectations, the three selected ELK3 KD cell lines showed an epithelial phenotype (reduced migration and invasive capacity, and increased adhesion), whereas control cells (C1) retained all the metastatic features of invasive MDA-MB-231 cells (Figure 1E). These results suggest that ELK3 KD has epithelial characteristics and a less invasive phenotype. Microarray analysis revealed that stable suppression of ELK3 in MDA-MB-231 cells led to the downregulation and upregulation of 1,081 and 1,339 genes, respectively (fold change > 4, GSE83325). Taken together, these results suggest that suppressing ELK3 reprograms MDA-MB-231 cells such that they display less invasive, epithelial characteristics.

### Suppression of ELK3 *in vivo* reprograms MDA-MB-231 cells to display a less invasive phenotype

**Figure 2:**
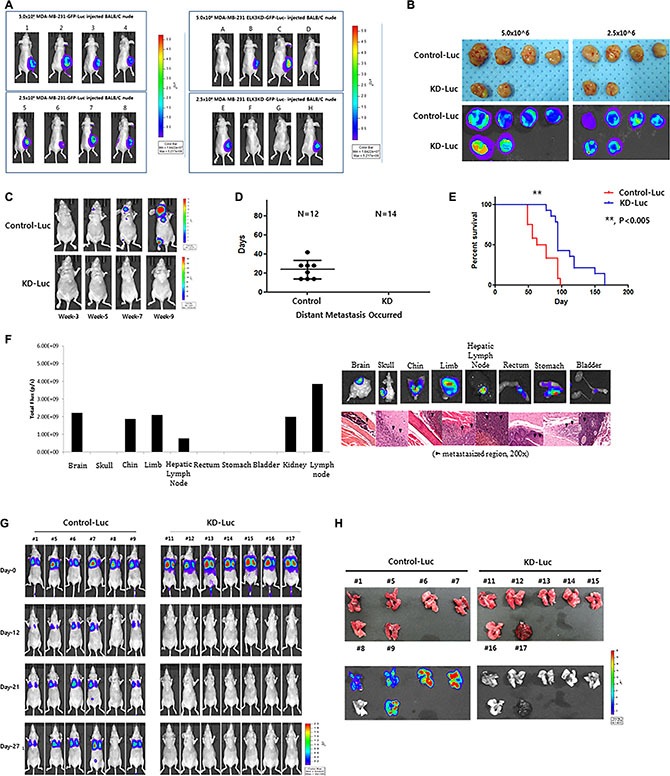
Knockdown of ELK3 expression in MDA-MB-231 cells results in loss of metastatic ability *in vivo* (**A**) Bioluminescence images of mice bearing tumors derived from subcutaneous injection of 2.5 × 10^6^ or 5.0 × 10^6^ Control-Luc or ELK3 KD-Luc cells. (**B**) *Ex vivo* bioluminescence images of tumors from all mice in each group. (**C**) Representative bioluminescence images showing progression of distant metastasis after cardiac injection of Control-Luc and ELK3 KD-Luc. Control-Luc (*n* = 12 mice; 1.0 × 10^5^ cells per mouse) and ELK3 KD-Luc (*n* = 14; 1.0 × 10^5^ cells per mouse) were injected into the left ventricle, and bioluminescence images were taken weekly. (**D**) Graph demonstrating the total number of mice with distant metastases post-cardiac injection, and the day of metastatic tumor occurrence. (**E**) Kaplan-Meier survival curves for mice injected with Control-Luc (red line) or ELK3 KD-Luc (blue line) cells. Statistical differences were evaluated using the log-rank test. ** *P* < 0.005. (**F**) Quantified bioluminescence image data. Data are derived from representative *ex vivo* bioluminescence images of metastasized organs (left panel). *Ex vivo* bioluminescence images of each organ isolated from the mice. Sections from each mouse organ were stained with hematoxylin and eosin (right panel). (**G**) Metastasis of Control-Luc and ELK3 KD-Luc cells was analyzed after tail vein injection. Control-Luc (*n* = 6 mice; 1.0 × 10^6^ cells per mouse) and ELK3 KD-Luc (*n* = 7 mice; 1.0 × 10^6^ cells per mouse) cells were injected into the tail vein, and success was confirmed by monitoring bioluminescent signals in the lungs at Day 0. Bioluminescence images were captured at the indicated times post-injection. (**H**) Lungs were isolated from each mouse at 4 weeks post-tail vein injection, and bioluminescence signals were monitored.

To analyze the tumorigenic and metastatic ability of ELK3 KD *in vivo*, we transplanted C1 and KD1 cells into the mammary fat pads of non-obese diabetic (NOD)/SCID mice. As shown in Figure 2A, all eight mice injected with 5 × 10^6^ or 2.5 × 10^6^ C1 cells developed tumors at 3 weeks post-inoculation, whereas only four of eight mice injected with KD1 cells developed tumors (Figure 2A). After injection of 2.5 × 10^6^ or 5.0 × 10^6^ C1 or KD1 cells, we observed that C1 tumors grew much faster than KD1 tumors (Figure S1). On the day of sacrifice, tumors were removed and measured: C1 tumors were significantly larger than KD1 tumors (Figure 2B).

We next monitored distant metastasis by intracardiac injection of C1 and KD1 cells, followed by measurement of luciferase activity in the tumor. Eight of the twelve mice injected with C1 cells (67%) showed distant metastasis, whereas none of the mice injected with ELK3 KD did so (Figure 2C and 2D). In the control group, experimental metastases were detected 24 (± 10) days (mean (SD)) after injection. The Kaplan-Meier survival curves for the C1 and KD1 groups revealed that KD1 mice survived longer than C1 mice (Figure 2E). Metastasized C1 cells were confirmed by hematoxylin and eosin (H&E) staining. Figure 2F showed that C1 cells metastasized to various organs, including the brain, limbs, and stomach.

When C1 and KD1 cells were injected into the tail, C1 cells colonized the lungs in 5/6 mice (83%), whereas 0/7 mice injected with KD1 cells showed lung metastasis (Figure 2G). Lungs from each mouse were carefully dissected, and bioluminescence imaging was performed. Consistent with the earlier data, the lungs from 5/6 (83%) C1 mice showed bioluminescence, whereas 0/7 (0%) lungs from KD1 mice did not (Figure 2H).

### Suppression of ELK3 in MDA-MB-231 cells disrupts the TGF-ß signaling pathway

**Figure 3:**
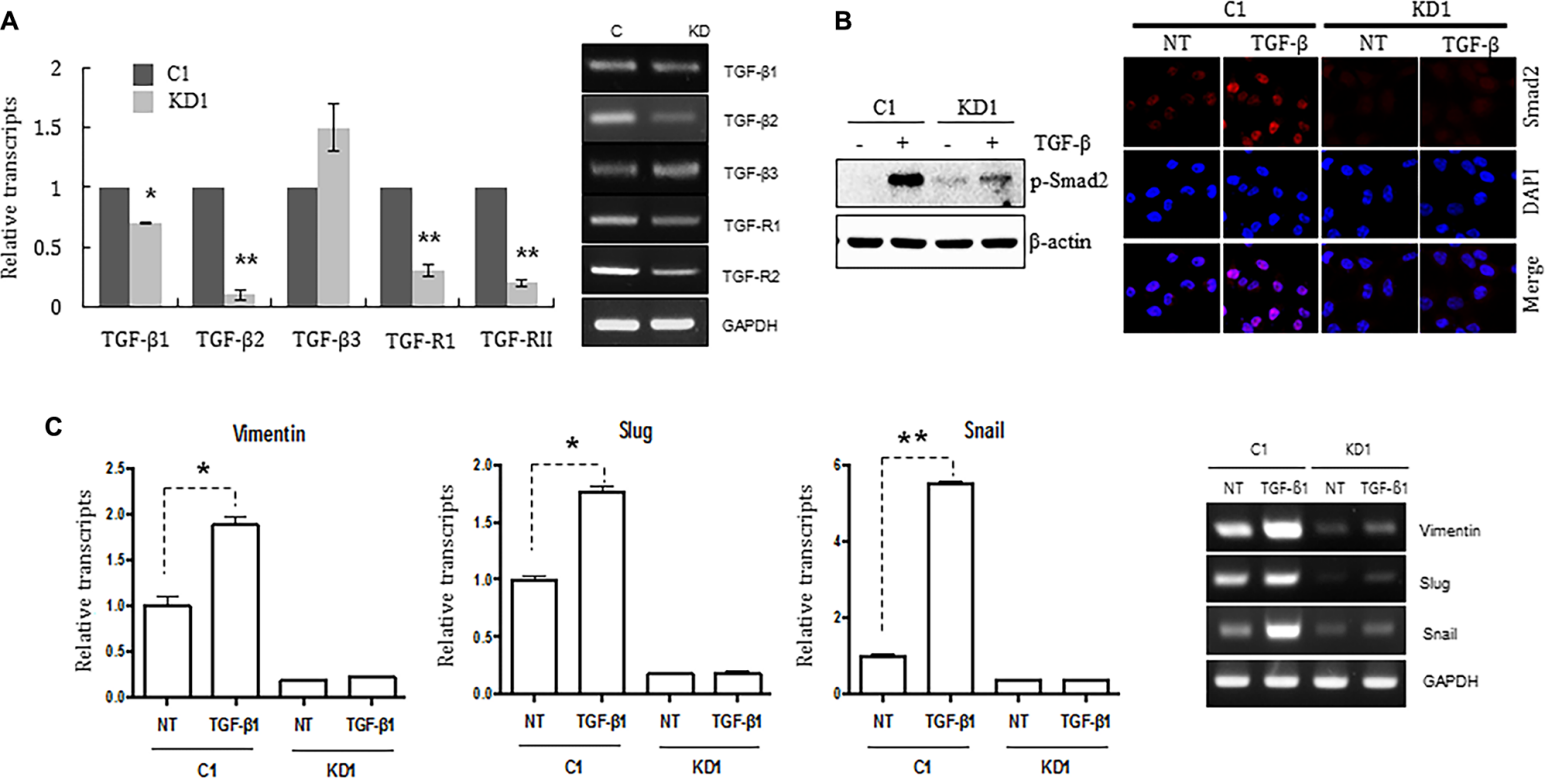
Knocking down ELK3 in MDA-MB-231 cells renders them unresponsive to TGF-ß (**A**) Relative expression of TGFß1, TGFß2, TGFß3, TGFR1, and TGFR2 in C1 and KD1 cells. (**B**) Phosphorylation and nuclear accumulation of Smad2 in C1 and KD1 cells upon TGF-ß treatment were analyzed by immunoblotting and immunocytochemical staining, respectively. (**C**) Relative expression of Vimentin, Slug, and Snail (target genes of Smad2) in C1 and KD1 cells treated with TGF-ß. Error bars represent the standard error from three independent experiments, each performed using triplicate samples. *P < 0.05 and **P < 0.01 (Student's *t*-test).

TGF-ß in the tumor microenvironment primes breast cancer cells to undergo EMT; metastasis to the lungs is particularly common [20–22]. Therefore, we postulated that TGF-ß signaling might not be functional in ELK3 KD cells. To test this, we examined the expression of TGF-ß receptors I and II (TGF-ß RI and RII), which are essential for transmission of the TGF-ß signal into cells. As expected, expression of TGF-ß RI and RII in ELK3 KD was less than 20% of that in control cells (Figure 3A). This suggests that many ELK3 KD cells are incapable of transmitting TGF-β signals. Indeed, immunoblot analysis of phospho-Smad2 expression, as well as immunostaining for nuclear localized Smad2 phosphorylation, revealed that Smad2 phosphorylation in ELK3 KD upon TGF-ß treatment was not significantly higher than that in C1 (Figure 3B). When Smad2 is phosphorylated and localized to the nucleus, it acts as a transcriptional activator that induces expression of EMT-related genes. Consistent with the phosphorylation status of Smad2 in ELK3 KD cells upon TGF-ß treatment, we found that the expression of mesenchymal markers such as Vimentin, Slug, and Snail in ELK3 KD cells in response to TGF-β was unchanged, whereas it increased by more than 2-fold in control cells (Figure 3C). Taken together, these data suggest that the TGF-ß signaling pathway is non-functional in ELK3 KD cells, which would explain, at least in part, the mechanism by which suppression of ELK3 deprives MDA-MB-231 cells of their metastatic characteristics.

### ELK3 indirectly and epigenetically suppresses GATA3 expression

**Figure 4:**
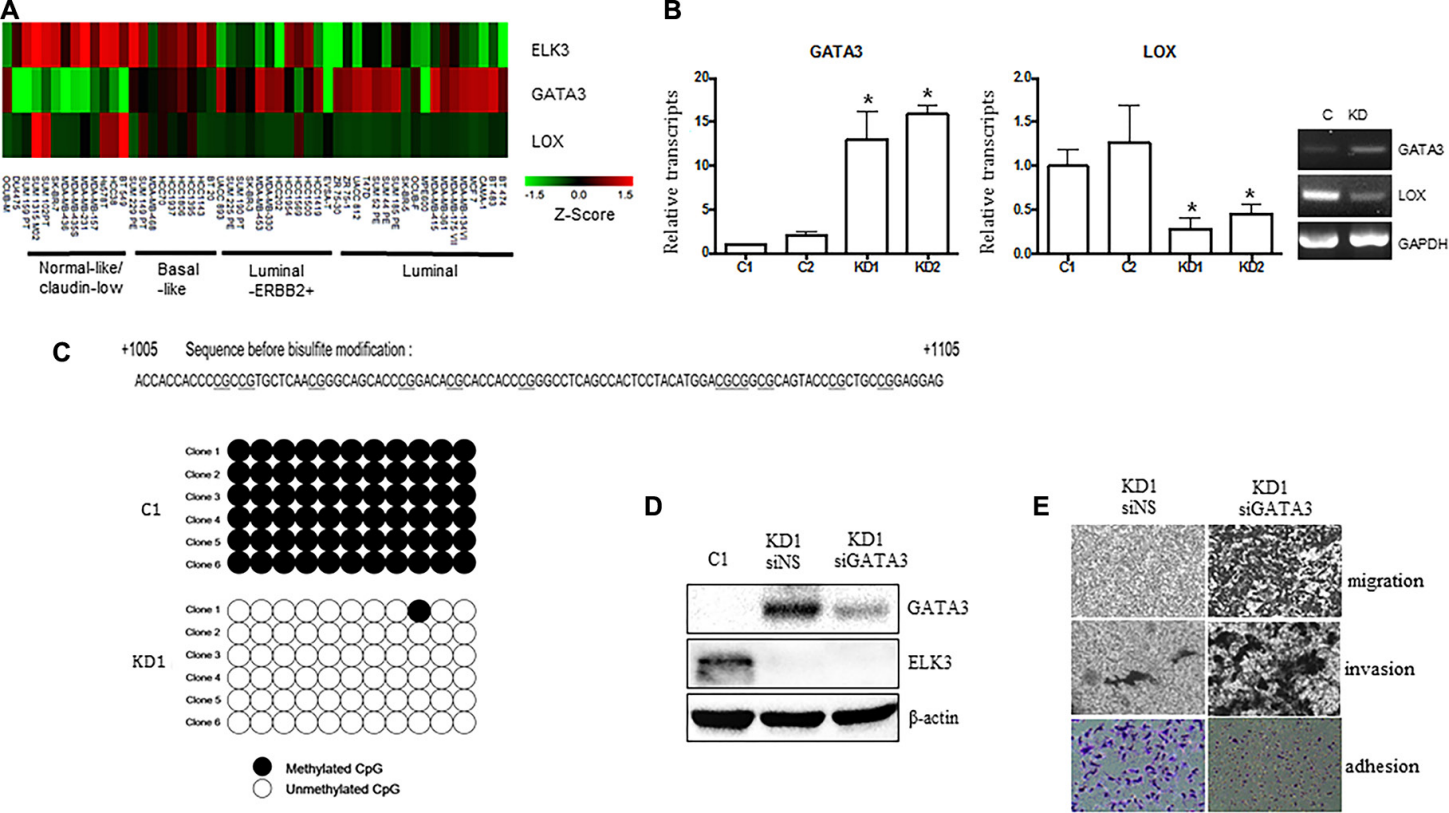
GATA3 orchestrates the phenotypic changes induced by suppression of ELK3 in MDA-MB-231 cells (**A**) Expression of ELK3, GATA3, and LOX, as shown on a heat map matrix derived from publicly available microarray data from 18 luminal, 12 luminal-ERBB2+, nine basal-like, 11 normal-like/claudin-low, and one other cell type [19]. (**B**) Relative expression of GATA3 and LOX in C1, C2, KD1, and KD2 cells. (**C**) Bisulfite sequencing analysis of the GATA3 promoter in C1 and KD1 cells. Each line reflects an independent clone. The white and black circles represent unmethylated and methylated CpG dinucleotides, respectively. (**D**) SiRNA-mediated suppression of GATA3 expression in KD1 cells. KD1 cells were transfected with a nonspecific siRNA (siNS) or with siRNA targeting GATA3 (siGATA3) for 48 h, and total cell extracts were analyzed by immunoblotting. (**E**) Migration, invasion, and adhesion of KD1 cells transfected with siNS or siGATA3 for 48 h (as described in Materials and methods). Error bars represent the standard error from three independent experiments, each performed using triplicate samples. *P < 0.05 (Student's *t*-test).

Recent studies report that overexpression of GATA3 in MDA-MB-231 (GATA3 OE) cells reprograms them from a basal to a luminal phenotype, which is characterized by reduced tumorigenesis and metastasis [14, 23, 24]. GATA3 also inhibits TGF-ß-dependent transcriptional responses in MDA-MB-231 cells and promotes MET [25]. In addition, GATA3 inhibits metastasis of MDA-MB-231 cells by suppressing lysyl oxidase (LOX), a metastasis-promoting matrix remodeling protein, via methylation of the LOX promoter [23]. Since the features of ELK3 KD cells were similar to those of GATA3 OE cells, we hypothesized that ELK3 is an upstream regulator of the GATA3-LOX axis in MDA-MB-231 cells. Therefore, we examined expression of ELK3, GATA3, and LOX by constructing a heat map matrix using public microarray data derived from breast cancer cell lines [19]. As expected, the expression of ELK3 was inversely correlated with that of GATA3 and LOX (Figure 4A). Consistent with this, we found that GATA3 expression was low in MDA-MB-231 cells that expressed high levels of ELK3.

We next examined whether GATA3 is indeed overexpressed (and LOX suppressed) in ELK3 KD cells. As expected, expression of GATA3 in ELK3 KD cell lines was high (Figure 4B). However, transient siRNA-mediated suppression of ELK3 expression in MDA-MB-231 cells did not increase GATA3 expression (Figure S2). This indicates that transcription of GATA3 is not directly increased by ELK3. One possible mechanism underlying GATA3 activation in ELK3 KD cells is epigenetic modification of the promoter. Indeed, bisulfite sequencing revealed that 11 CpG sequences within the GATA3 promoter were completely demethylated in ELK3 KD cells (Figure 4C). We next examined whether the loss of metastatic features from ELK3 KD cells occurred via activation of GATA3. When overexpression of GATA3 in ELK3 KD cells was suppressed by siRNA transfection, we observed a significant increase in the migration and invasion of the cells in a Matrigel assay (Figure 4D and 4E). In addition, the increase in cell adhesion observed upon suppression of ELK3 was reversed by suppressing GATA3 (Figure 4E). Despite the finding that the proliferation rate of ELK3 KD cells was higher than that of control cells, changes in the pH of the medium occurred more slowly than in that of control cultures (Figure S3A). Consistent with this, the seahorse XF cell energy phenotype revealed that both the oxygen consumption rate (OCR) and the extracellular acidification rate (ECAR) increased in ELK3 KD when compared with control cells (Figure S3B). When GATA3 expression in ELK3 KD was suppressed by siRNA, both OCR and ECAR fell to levels observed in control cells (Figure S3C). Taken together, these data suggest that suppression of ELK3 results in epigenetic activation of the GATA3 promoter to induce GATA3 expression. Thus, overexpression of GATA3 in ELK3 KD cells is the main factor that drives the reprogramming of MDA-MB-231 cells to a non-invasive luminal phenotype.

## DISCUSSION

Induction of tumor metastasis is accompanied by dynamic reprogramming of the epigenome, a process that involves changes in DNA methylation and histone modification. After invading distant organs, metastasized tumor cells undergo reversal of EMT (referred to as MET) at the metastatic site to enable colonization [26, 27]. This implies that epigenetic plasticity is a central regulator of tumor dissemination and re-localization. This model is supported by recent observations that circulating breast cancer cells exhibit dynamic changes in epithelial and mesenchymal features [28]. Here, we showed that suppressing ELK3 expression leads to the transdifferentiation of MDA-MB-231 cells *in vitro* and *in vivo*, resulting in the loss of metastatic characteristics. Major EMT-inducing transcription factors, such as Slug and Snail, in ELK3 KD cells were not transcriptionally activated by TGF-ß. ELK3 KD-derived tumors in the xenograft model were smaller than control tumors, despite that the proliferation rate of ELK3 KD cells was higher than that of control MDA-MB-231 *in vitro*. A possible mechanism underlying the discrepancy between *in vitro* proliferation and *in vivo* tumor formation is that ELK3 KD show reduced secretion of proteins that communicate with the microenvironment and drive tumor development. Indeed, the secretome of ELK3 KD was significantly different from that of control cells; for example, we noticed that the secretion of angiogenic factors such as VEGF by ELK3 KD was reduced by about ∼50% (Figure S4A). In addition, the expression of CD31, a marker of microvessel formation, was significantly lower in ELK3 KD tumors compared to control tumor (Figure S4B, S4C). Thus, we speculate that alterations to the ELK3 KD secretome are related to the formation of smaller tumor masses in the xenograft experiments.

The extensive changes in gene expression profile caused by suppression of ELK3 led us to identify GATA3 as a critical regulator of metastatic changes in ELK3 KD cells. We found that transfecting ELK3 KD cells with siGATA3 restored their migratory and invasive characteristics. Based on these results, we suggest that the ELK3-GATA3 axis is a central and crucial regulatory component of EMT/MET. ELK3 is thought to function as a transcriptional activator in MDA-MB-231 cells, which harbor a highly active RAS/ERK signaling pathway. Overexpression of GATA3 in ELK3 KD cells indicates that GATA3 is not a direct target for ELK3. Demethylation of the GATA3 promoter in ELK3 KD cells suggests that epigenetic factors that promote DNA demethylation are activated in MDA-MB-231 cells upon suppression of ELK3. Indeed, the DNA demethylase, TET1, suppression of which is essential for KRAS-mediated DNA hypermethylation and malignant transformation [29], was highly expressed in ELK3 KD cells, whereas expression of DNMT3A and DNMT3B was similar to that in control cells (Figure S5).

A recent study demonstrated that both OCR, an indicator of oxidative phosphorylation, and ECAR increase in low-invasive cancer cells [30]. Changes in the OCR and ECAR in ELK3 KD and siGATA3-transfected ELK3 KD cells imply that comprehensive metabolic reprogramming in MDA-MB-231 cells is accompanied by expression of ELK3 and GATA3.

Despite our finding that a non-functional TGF-ß signaling pathway (resulting from suppression of TGF-ß R1/RII) in ELK3 KD cells might contribute to a less metastatic phenotype, transfection of siGATA3 did not rescue the TGF-ß response of ELK3 KD (Figure S6). Thus, it remains unclear whether TGF-ß R1/RII is epigenetically suppressed in ELK3 KD.

The findings described herein indicate that ELK3 may play a previously unrecognized and important role in breast cancer metastasis based on data suggesting that its loss of function aids reprogramming of basal types of breast cancer cells to a less invasive, epithelial cell phenotype. Suppression of ELK3 in another basal type breast cancer cell line, Hs578T, also accompanied with GATA3 activation as well as reduced migration and invasive capacity *in vitro* (Figure S7). A recent report shows that ELK3 drives malignancy in SCCs [8], suggesting that the role of ELK3 as a promoter of metastasis is not likely to be limited to breast cancer. Further studies should focus on identifying genes directly targeted by ELK3 and on furthering our understanding of the metastatic mechanisms that it regulates. This may facilitate identification of effective therapeutic targets.

## MATERIALS AND METHODS

### Cell culture and knockdown of ELK3 and GATA3

MDA-MB-231-GFP-Luc cells were purchased from Perkin Elmer (Boston, MA, USA) and cultured in Dulbecco's Modified Eagle's Medium (DMEM) supplemented with 10% fetal bovine serum (FBS) (Gibco BRL). ShRNA plasmids targeting human ELK3 (catalog no. RHS4531-EG2004) or human GATA3 (catalog no. RHS4531-EG2625) were purchased from Dharmacon. ELK3 KD breast cancer cell lines (KD1, KD2, and KD3) and two control (C) cell lines (C1 and C2) were established by infecting MDA-MB-231-GFP-Luc cells with a retrovirus generated from control or shRNA retroviral plasmids containing ELK3 (catalog no. RHS4531-EG2004). Retroviral shRNA targeting GATA3 (catalog no. RHS4531-EG2625) was transfected into ELK3 KD MDA-MB-231 cell lines.

### Microarray analysis

Total RNA was extracted using TRIzol (Invitrogen), and biotinylated cRNA were prepared from 0.55 μg of total RNA using the Illumina TotalPrep RNA Amplification Kit (Ambion, Austin, TX), according to the manufacturer instructions. Following fragmentation, cRNA (1.5 μg) was hybridized to the Illumina HumanHT-12 v4 Expression BeadChip, according to the manufacturer's instructions (Illumina). Arrays were then scanned with an Illumina Bead Array Reader Confocal Scanner, according to the manufacturer's instructions. Array data processing and analysis were performed using Illumina BeadStudio v3.1.3 (Gene Expression Module v3.3.8). The microarray data is deposited in the Gene Expression Omnibus data repository (GEO: GSE83325).

### Cell adhesion, migration, and invasion assays

A standard crystal violet adhesion assay was performed as previously described [31]. For the migration assay, MDA-MB-231 cells (5 × 10^3^) were added to each upper well of a Transwell plate (Corning Inc, Corning, NY, USA). The lower portion of the well contained DMEM supplemented with 10% FBS as a chemoattractant. After incubation for 24 h, cells that migrated to the bottom chamber were removed from the underside of the membrane, fixed in 4% paraformaldehyde (Santa Cruz), and stained with crystal violet. For the invasion assays, cells (8 × 10^4^) were placed onto a Transwell membrane coated with 50 ml of Matrigel (BD Biosciences) and then incubated for 36 h. Migrated cells were processed as described above.

### RNA extraction and real-time RT-PCR

Total RNA was extracted using TRIzol (Invitrogen), and 2–5 μg was reverse-transcribed using the SuperScriptII™ First-Strand Synthesis System (Invitrogen), according to the manufacturer's instructions. Real-time RT-PCR was performed using the resulting cDNAs and the Quantitect SYBR Green PCR kit (Qiagen). Expression of target genes was normalized against that of glyceraldehyde 3-phosphate dehydrogenase (GAPDH). The PCR primer pairs (sense and antisense) are listed in Table S1.

### Western blot analysis

Cells were washed with PBS and lysed in tissue lysis buffer (20 mM Tris-base, pH 7.4, 137 mM NaCl, 2 mM EDTA, 1% Triton X-100, 25 mM β-glycerophosphate, 2 mM sodium pyrophosphate, 10% glycerol, 1 mM sodium orthovanadate, 1 mM benzamidine, and 1 mM phenylmethysulfonyl fluoride). Total cell extracts were resolved by sodium dodecyl sulfate-polyacrylamide gel electrophoresis, transferred to Immobilon-P membranes (Millipore, Bedford, MA, USA; http://www.Millipore.com), and blotted with antibodies against ELK3 (sc-17860), Smad2 (sc-8332, Santa Cruz Biotechnology), phospho-Smad2 (#3101, Cell Signaling Technology), and GATA3 (sc-268, Santa Cruz Biotechnology). Immunoreactivity was detected by enhanced chemiluminescence (ECL; Amersham).

### Xenograft mouse model

All animal experiments were approved by the Institutional Animal Care and Use Committee (IACUC) of National Cancer Center. Four-week-old BALB/c nude mice were purchased from Orient Bio. (Republic of Korea) and housed at the experimental animal facility at the National Cancer Center. A tumor xenograft model was established when the mice reached 5 weeks of age. Mice were randomly assigned to four different groups and injected with the following: control-Luc cells (2.5 × 10^6^ or 5.0 × 10^6^) or ELK3 KD-Luc cells (2.5 × 10^6^ or 5.0 × 10^6^). Tumor size and the weight of the mice were measured three times per week until the day of sacrifice. For IVIS imaging, D-Luciferin (Perkin Elmer, USA) was injected (intraperitoneally) into mice using a 1 ml BD Syringe Leuer-Lok Tip fitted with a 26G 1/2 BD PrecisionGlide Needle (BD Korea, Republic of Korea). Mice were imaged 8 min later.

### *In vivo* metastasis: cardiac injection model

BALB/c nude mice (4 weeks old) were purchased from Orient Bio. For the metastasis model, mice received cells via cardiac injection through the left ventricle. Metastasis was monitored weekly by IVIS Lumina III XR imaging. On the day of sacrifice, IVIS imaging was performed before organs were harvested; samples were then mounted in paraffin blocks. Total flux (p/s) within each organ was measured using the IVIS Lumina III XR imaging system, and data were analyzed using Living Image 2.60.1 (PerkinElmer, USA) software. This study was reviewed and approved by the IACUC of National Cancer Center Research Institute (NCCRI), Goyang-si, Gyeonggi-do, Republic of Korea. NCCRI is an Association for Assessment and Accreditation of Laboratory Animal Care International (AAALAC International, no. 001392)-accredited facility and abides by the Institute of Laboratory Animal Resources (ILAR) guidelines.

### *In vivo* metastasis: tail vein injection model

BALB/c nude mice (4 weeks old) were obtained from Harlan laboratories (DooYeol Biotech, Republic of Korea) and divided into two groups: Control-Luc and ELK3 KD-Luc. Tail vein injection was performed 1 week after the mice reached 5 weeks of age. Cells were injected into the tail vein using a 1 ml BD syringe fitted with a 31G Ultra-Fine II short needle (BD Korea, Republic of Korea). The success of the tail vein injection was evaluated immediately by IVIS Lumina III XR imaging, and metastasis was monitored weekly. Bodyweight was measured twice a week until the day of sacrifice (at 4 weeks post-tail vein injection). Samples were prepared as described above.

### Histology

Organs containing metastatic deposits showing luciferase expression were harvested and stabilized in 10% Neutral Buffered Formalin (BBC biochemical, USA). Sections of paraffin-embedded tissues were stained with hematoxylin (Merck, USA) and eosin (Merck, USA) and examined under a ScanScope XT virtual digital slide scanner (Aperio, USA) (magnification, ×200). The magnified slide was visualized using Imagescope software version 11.2.0.782 (Aperio, USA). Sections of paraffin-embedded organs containing metastatic deposits were examined by immunofluorescence using a Zeiss AxioImager M1 Fluorescence microscope (Carl Zeiss, Germany).

### Bisulfite pyrosequencing

Bisulfate treatment and DNA cleanup were performed using the EpiTect 96 Bisulfite Kit, according to the manufacturer's instructions. Sodium bisulfite-modified genomic DNA from ELK3 KD or control cells was amplified by PCR using primers GATA3-F (5′-GTT GGG TGA GTT ATT ATT ATT T-3′) and GATA3-R (5′-ATT AAA AAA CAC ATC CAC CTC CT-3′). The PCR products were then cloned into the T-vector (Takara) and sequenced.

### Statistical analysis

All statistical analyses were performed using GraphPad Prism 5 (GraphPad Prism, La Jolla, CA, USA). Data were analyzed using Student's *t*-test. Differences were considered statistically significant at *P* < 0.05.

## SUPPLEMENTARY MATERIALS FIGURES AND TABLE


