# Supplementary material for: The ELK3-GATA3 axis orchestrates invasion and metastasis of breast cancer cells in vitro and in vivo
Source: Oncotarget. 2016 Aug 19;7(40):65137–46. doi: 10.18632/oncotarget.11427 (PMC5323143; doi:10.18632/oncotarget.11427)
Supplement: Supplementary file 1 [file oncotarget-07-65137-s001.pdf]

The ELK3-GATA3 axis orchestrates invasion and metastasis of breast cancer cells *in vitro* and *in vivo*

Supplementary Materials

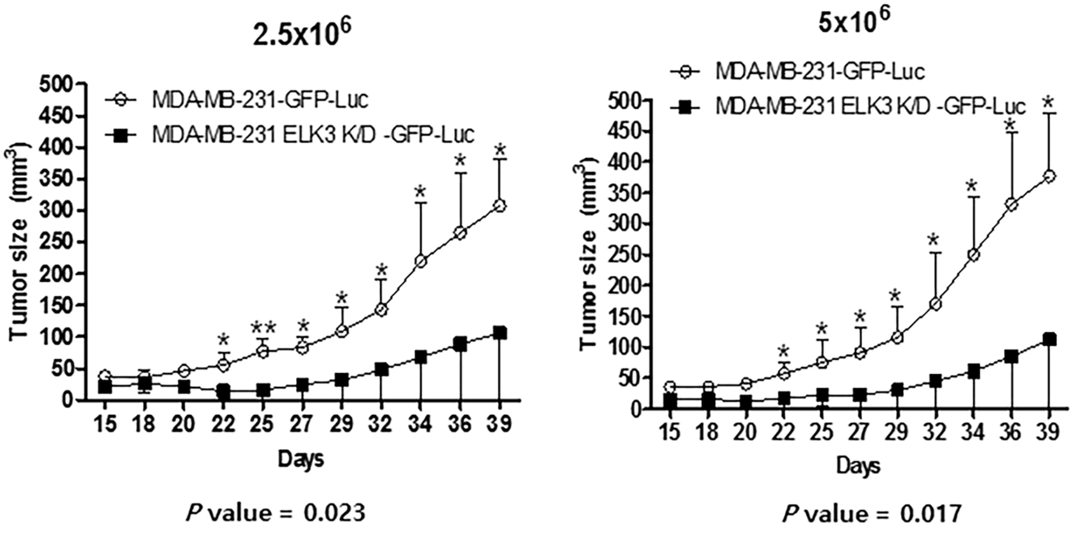

Supplementary Figure S1: Tumor growth of control and ELK3 KD *in vivo*. After injection of 2.5×10^6 or 5.0×10^6 C1 or KD1 cells into BALB/c nude mice, tumor size was measured at the indicated days after xenograft. $^*P < 0.05$ (Student's *t*-test).

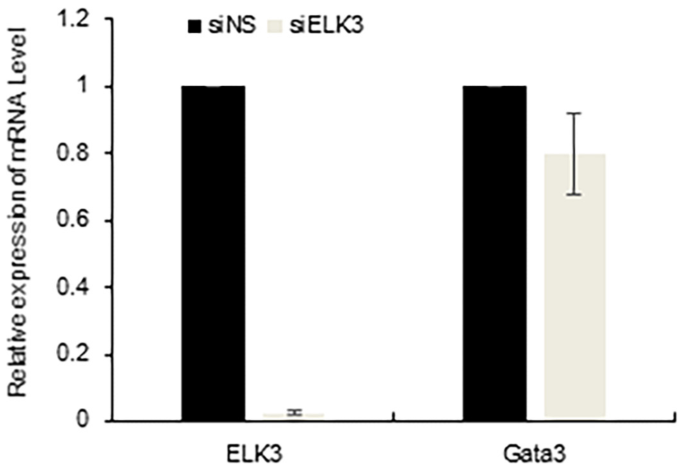

Supplementary Figure S2: The effect of transient suppression of ELK3 on the GATA3 expression of MDA-MB-231.

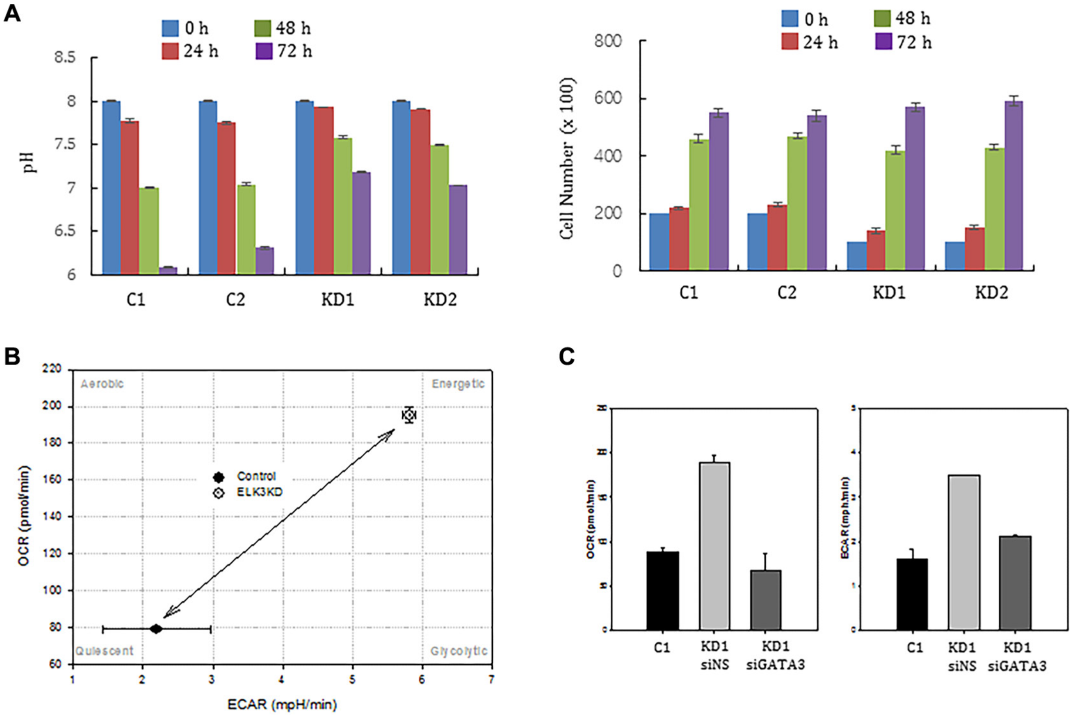

Supplementary Figure S3: Suppressing ELK3 alters the metabolism of MDA-MB-231 cells. (**A**) The pH of the culture medium from C1, C2, KD1, and KD2 cells was analyzed at the indicated times after plating (left panel). C1 and C2 (2×10^4) or KD1 and KD2 (1×10^4) cells were plated, and numbers were counted at the indicated times (right panel). (**B**) Seahorse XF cell energy phenotype of C1 and KD1 cells. Suppression of ELK3 induced a metabolic switch in MDA-MB-231 cells. OCR and ECAR in KD1 cells were higher than in C1 cells. (**C**) Seahorse XF cell energy phenotype of KD1 cells transfected with nonspecific siRNA (siNS) or with siRNA targeting GATA3 (siGATA3). OCR and ECAR in KD1 cells were reduced upon suppression of GATA3.

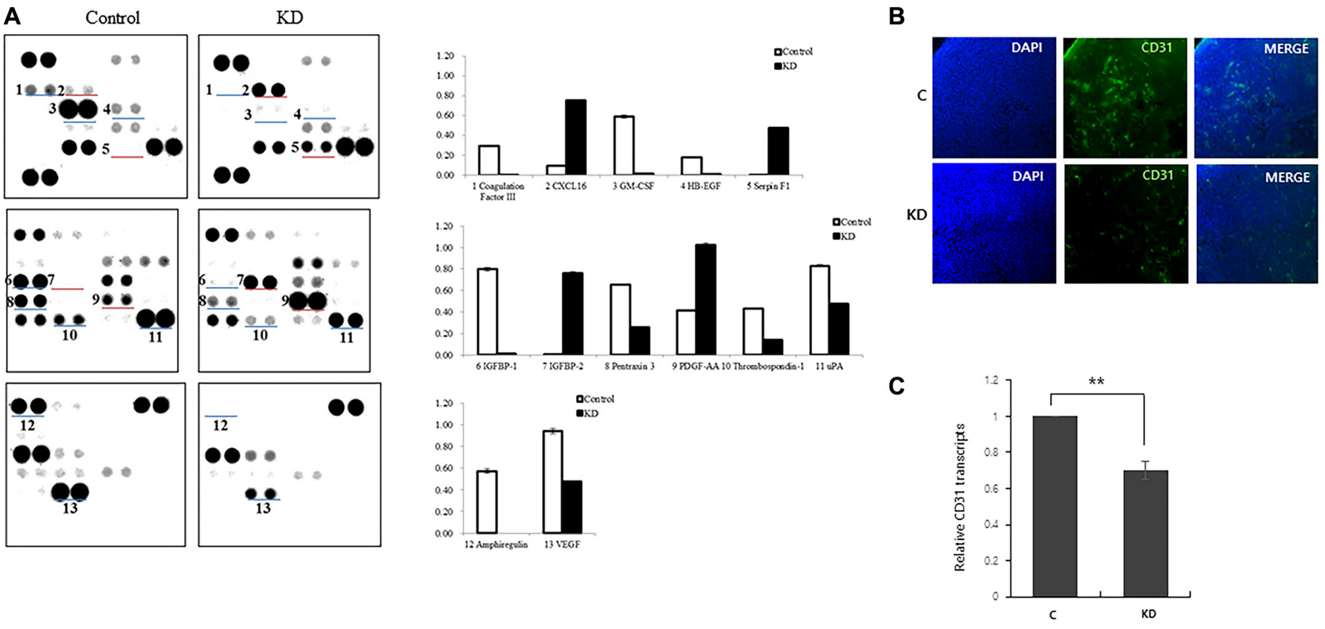

Supplementary Figure S4: Suppressing ELK3 alters the expression of angiogenesis-related proteins in MDA-MB-231 cells. (**A**) The secretome of C1 and KD1 was analyzed with a "Proteome Profiler Array-Human Angiogenesis Array Kit" (ARY007, R&D systems). For quantitative analysis, the density of each dot was measured using Multi Gauge V3.0 software. CD31 expression in the tumors of C1 and KD1 was analyzed by immunohistochemical staining (**B**) and realtime RT-PCR analysis (**C**).

SYMBOL	DEFINITION	ACCESSION	fold change (KD/C)
TET1	Tet methylcytosine dioxygenase 1	NM_030625.2	13.72
DNMT1	DNA (cytosine-5-)-methyltransferase 1	NM_001379.2	-1.62
DNMT3A	DNA methyltransferase 3A	NM_007872.4	-1.05
DNMT3B	DNA methyltransferase 3B	NM_006892.3	1.37

Supplementary Figure S5: The microarray result of Tet1, DNMT1, DNMT3A and DNMT3B expression in the ELK3KD and control cells were listed in the table.

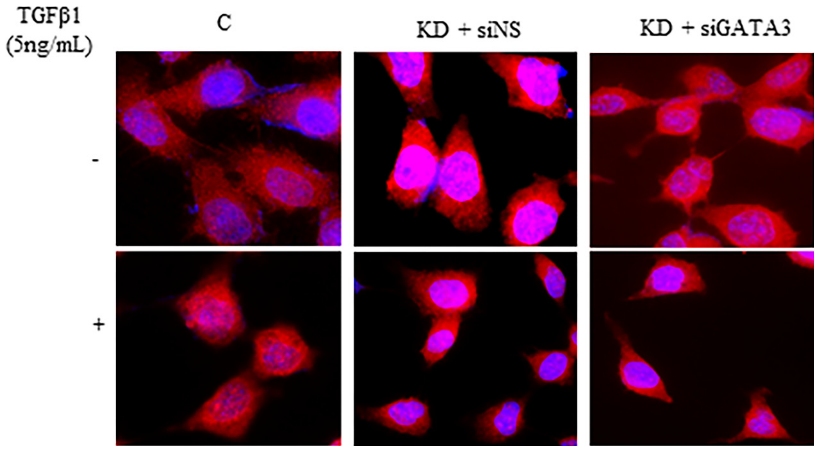

Supplementary Figure S6: The effect of TGFß1 treatment on the nuclear translocation of Smad2 in the control, ELK3 KD and ELK3KD transfected with siGATA3.

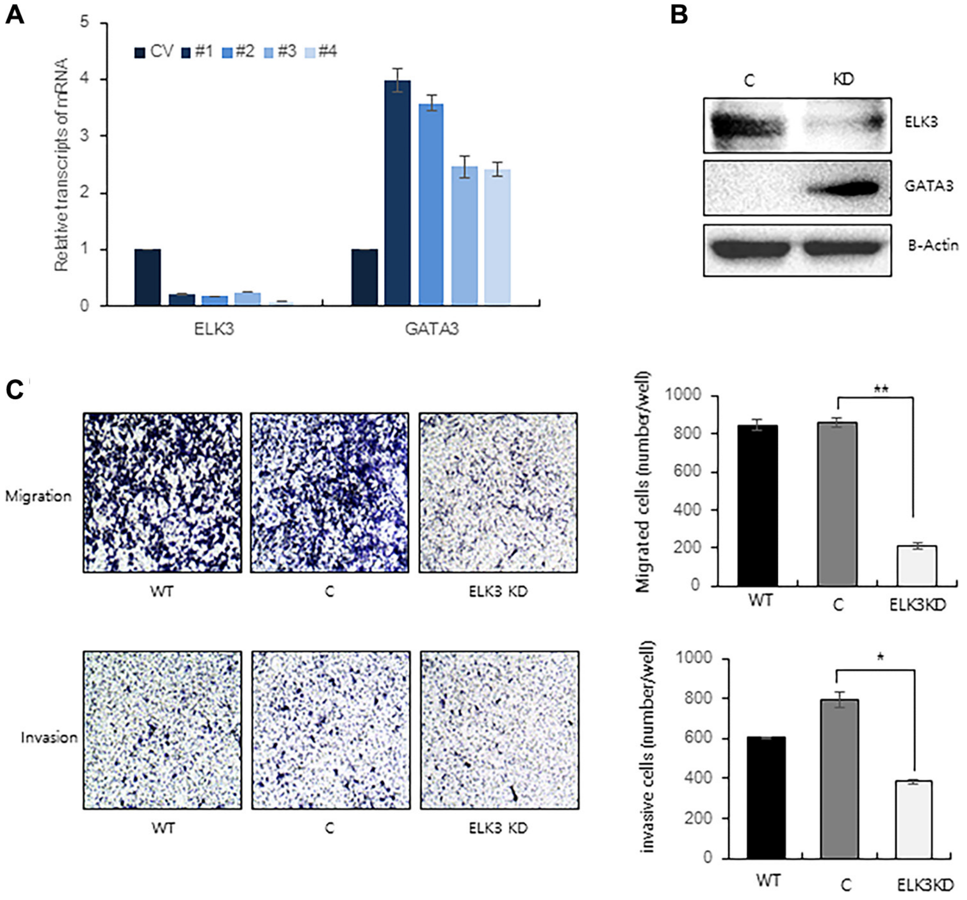

Supplementary Figure S7: The effect of ELK3 suppression on the GATA3 expression, invasion and migration of Hs578T. (**A**) Relative expression of GATA3 was analyzed by realtime RT-PCR in the four ELK3 knockdowned cell lines of Hs578T. ELK3 was stably suppressed by the chromosomal integration of ELK3 targeting shRNA. (**B**) ELK3 and GATA3 expression in the control and ELK3KD was analyzed by immunoblot analysis (**C**) Migration and invasion were analyzed as described in Materials and methods. Error bars represent the standard error from three independent experiments, each performed using triplicate samples. *$P < 0.05$ and **$P < 0.01$ (Student's *t*-test).

Supplementary Table S1: PCR primer sequences

Gene	Primer sequence (5′to 3′)
GAPDH	F-ACC ACA GTC CAT GCC ATC AC
	R-TCC ACC ACC CTG TTG CTG TA
ELK3	F-TGT ATG CTG GAG AGC AGT GG
	R-ACC CAA AGG CTT GGA AAT CT
TGFß1	F-CAG AAA TAC AGC AAC AAT TCC TGG
	R-TTG CAG TGT GTT ATC CCT GCT GTC
TGFß2	F-TCA AGA GGG ATC TAG GGT GGA A
	R-GGC ARG CTC CAG CAC AGA A
TGFß3	F-GGA GGA GGA GAA GGA GGA GA
	R-CAA ATG CCC AAC TCA TTG TG
TGFR1	F-CCT CTA GAG AAG AAC GTT CGT
	R-GGC TTT CCT TGG GTA CCA ACA A
TGFR2	F-GGT CGC TTT GCT GAG GTC TA
	R-CTT GAG GTC CCT GTF CAC GAT
Vimentin	F-GAG AAC TTT GCC GTT GAA GC
	R-GCT TCC TGT AGG TGG CAA TC
Slug	F-GGG GAG AAG CCT TTT TCT TG
	R-TCC TCA TGT TTG TGC AGG AG
Snail	F-CCT CCC TGT CAG ATG AGG AC
	R-CCA GGC TGA GGT ATT CCT TG
GATA3	F-GCG GGC TCT ATC ACA AAA TGA
	R-GCC TTC GCT TGG GCT TAA T
LOX	F-CAG GGT GCT GCT CAG ATT TCC
	R-GGT AAT GTT GAT GAC AAC TGT GC
